# Integrated multi-omics analysis reveals the immunotherapeutic significance of tumor cells with high FN1 expression in ovarian cancer

**DOI:** 10.3389/fmolb.2025.1611964

**Published:** 2025-06-19

**Authors:** Xinyi Zhang, Zhikai Xiahou, Fu Zhao, Qing Wu, Wei Nie, Shouyan Wang

**Affiliations:** ^1^ Clinical Medical College, Southwest Medical University, Luzhou, China; ^2^ China Institute of Sport and Health Science, Beijing Sport University, Beijing, China; ^3^ School of Traditional Chinese Medicine, Jinan University, Guangzhou, China; ^4^ Dongying People’s Hospital (Dongying Hospital of Shandong Provincial Hospital Group), Dongying, Shandong, China

**Keywords:** FN1 signaling pathway, prognostic stratification, spatial transcriptomics, immune infiltration analysis, ovarian cancer

## Abstract

**Background:**

Ovarian cancer is a highly lethal gynecological malignancy characterized by significant heterogeneity and immunosuppressive tumor microenvironments, contributing to poor prognosis and therapeutic resistance. This study investigates the immunological and prognostic significance of FN1-expressing tumor cells using integrated multi-omics approaches.

**Methods:**

The study used GEO database data processed with Seurat and Harmony R. Each cluster had marker genes and cells were tested for preference. Cell stemness was measured using AUCell and CytoTRACE. The gene regulatory network was analyzed using pySCENIC. Molecular signaling exchange study was done with CellChat. And immune infiltration as well as prognostic stratification was performed using bulk analysis. Finally, the identified FN1 targets were validated in conjunction with the spatial transcriptome as well as experimentally.

**Results:**

The study highlighted FN1 expression as a key factor in ovarian cancer prognosis and immune resistance. High FN1 tumor cells were linked to poor survival. FN1 knockdown inhibited tumor growth by reducing tumor cells aggregation, invasion, and migration. Our findings suggested that FN1+ tumor cells contributed to immunotherapy resistance, making FN1 a potential biomarker and therapeutic target for improving treatment outcomes in ovarian cancer.

**Conclusion:**

A prognostic model created based on FN1 tumor cells provided a new idea for clinical staging of ovarian cancer patients. Meanwhile, this study provided new insights into the heterogeneity of tumor cells and suggested a potential therapeutic target, FN1, which could be helpful for precise immunotherapy of ovarian cancer.

## 1 Introduction

Ovarian cancer, a very lethal gynecologic malignancy, is characterized by its intricate tumor microenvironment (TME) and its metastatic potential (Deng et al., 2022; Almeida-Nunes et al., 2022; Zhu et al., 2022). Ovarian cancer is a heterogeneous disease characterized by a variety of tumors that exhibit diverse clinicopathological, genetic, and prognostic features, demonstrating significant tumor heterogeneity both within and among subtypes (Kossai et al., 2018; Xiao et al., 2022; Cho and Shih, 2009). High-grade plasmacytoid carcinoma represents the most common subtype of ovarian cancer, characterized by its aggressive nature and generally poor prognosis for affected patients (Wang Y. et al., 2022). Epithelial ovarian cancer, particularly high-grade serous ovarian carcinoma, characterized by extensive genomic instability, high rates of intraperitoneal metastasis, and frequent development of chemoresistance. Surgery and chemotherapy are the primary treatments for ovarian cancer; however, chemotherapy resistance leads to patients’ recurrence within a few years of initiating treatment (Wood and Ledermann, 2022; Yang et al., 2020; Jiang et al., 2020). Despite initial responses to cytoreductive surgery and platinum-based chemotherapy, the majority of patients experience relapse, often within 18 months, due to acquired resistance and the persistence of tumor subclones (Dou et al., 2023). Immunotherapy has grown rapidly in the past 20 years, modernizing cancer treatment and ushering in precision healthcare (Morand et al., 2021; Kuroki and Guntupalli, 2020; Zhang L. et al., 2024; Ye et al., 2025). The mechanisms underlying immune evasion remain inadequately understood, thereby constraining the efficacy of cancer immunotherapy. Recent advances in molecular profiling have revealed that ovarian cancer is not a single disease but rather a heterogeneous group of neoplasms with distinct molecular, cellular, and clinical characteristics. This heterogeneity is further complicated by the TME, which plays a pivotal role in immune evasion, therapeutic resistance, and disease progression. Emerging immunotherapeutic strategies, including immune checkpoint inhibitors and adoptive T cell therapies, have shown limited efficacy in unselected ovarian cancer populations. A deeper understanding of immune-tumor interactions and the identification of key immunomodulatory drivers are urgently needed to improve patient stratification and treatment outcomes (Xu et al., 2020). In this context, our study focuses on FN1+ tumor cells using an integrated multi-omics approach, aiming to elucidate their role in immune escape and prognostic stratification. This work contributes to the broader effort to develop precision immunotherapies tailored to the complex immunobiology of ovarian cancer.

Single-cell RNA sequencing (scRNA-seq) is a powerful method for examining ovarian cancer heterogeneity with unprecedented detail (Xu et al., 2022; Zhang et al., 2023a; Zhao et al., 2025a; Zhao et al., 2025b). This method enables researchers to delineate diverse cell types, ascertain biological states, and reveal dynamic interactions within the tumor microenvironment by examining individual cells (Olbrecht et al., 2021; Olalekan et al., 2021). The TME strongly influences tumor growth, progression, and therapy response (Yu et al., 2023; Han et al., 2023). In the TME, immune cells and ovarian cancer cells engage in reciprocal signaling that modifies the immune response and influences the progression of the disease (Luo et al., 2021). Ovarian cancer with TME has been shown to recruit several immune cell types, and recent years have yielded a more profound understanding of the intricacies of their interactions (So et al., 2018; Lin S. C. et al., 2022; Kasikova et al., 2024). The TME of ovarian cancer is recognized for its significant immunosuppressive properties, facilitating evasion of immune surveillance and unrestricted tumor proliferation (Cai and Jin, 2017). Consequently, it is essential to deepen our comprehension of the underlying mechanisms to formulate enhanced tactics and augment the clinical applicability of immunotherapy.

Spatial Transcriptomics (ST) is a technology that examines and delineates the expression profiles of specific cell types in a spatial context to elucidate expression variations among organs, tissues, and pathological conditions, and it can resolve transcript profiles of tissues at distinct spatial locales (Rao et al., 2021). ST technology, when integrated with traditional single-cell sequencing, *in situ* methods, and other histological techniques, facilitates the examination of cellular heterogeneity and localization within tissue architecture. This approach offers a more accurate research trajectory for disease investigation, significantly enhancing the comprehension of pathogenic mechanisms and informing targeted therapeutic strategies.

In the current study, we dissected tumor-immune cell interactions in ovarian cancer to improve immunoprecision therapy and identify barriers to immunotherapy by using scRNA-seq and ST. Utilizing CellChat, we further examined intercellular communication within the tumor microenvironment and found FN1-CD44, a significant signaling network that may be targeted for immunoprecision treatments (Wan et al., 2024). We identified it as a significant mediator of interactions between tumor cells and stromal components, indicating its significance in establishing microenvironments favorable to tumor proliferation. This cellular communication may be the most effective intervention for ovarian cancer. Simultaneously, we illustrated the distribution of important subtypes and signaling pathways in tissue sections from ovarian cancer patients utilizing ST techniques, which have confirmed their viability as immunotherapeutic targets. Ultimately, *in vitro* assays for functional confirmation demonstrated that FN1 knockdown diminished tumor cell invasiveness and activity. These findings offer significant insights into the molecular mechanisms of ovarian cancer and pinpoint potential targets for personalized immunotherapy.

## 2 Methods

### 2.1 Origin of data

The GEO database (https://www.ncbi.nlm.nih.gov/geo/) supplied the ovarian cancer single-cell RNA sequencing dataset, obtained under accession number GSE181955. The samples comprised one normal ovary, two primary ovarian cancer specimens, and two omental tissues. Tissue slices for spatial transcriptomics were acquired from GSE211956. This study did not necessitate ethical approval as it utilized publicly available data.

### 2.2 RNA sequencing in single cells

The Seurat package (version 4.3.0) was used to process gene expression data in R (version 4.2.0) (Butler et al., 2018). 500 < nCount <100,000, 300 <nFeature <7,500, mitochondrial gene expression ≤25% of total genes, and erythrocyte gene expression ≤5% of total genes were the quality control criteria used to screen the cells. The data were first normalized using “NormalizeData” (Jiang et al., 2022), followed by “FindVariableFeatures” (Wu et al., 2021; Zhao et al., 2024a; Lin L. et al., 2024; Ni et al., 2025; Hou et al., 2025; Li et al., 2025; Wang et al., 2024) to select the first 2,000 variable genes, then followed by “ScaleData” to standardize the data. Next, we downscaled the obtained data using principal component analysis (PCA) and used the Harmony R package (version 0.1.1) to handle batch effects. Then, we used the “FindClusters” and “FindNeighbors” tools (Ge et al., 2024; Jin et al., 2024; Nie et al., 2024; Zhang Y. et al., 2024) to cluster the first 30 principal components (PCs) into cells. Lastly, based on the important PCs, gene expression was visualized using uniform manifold approximation and projection (UMAP) (Ge et al., 2024; Ding et al., 2023).

### 2.3 Cell type identification

To identify marker genes for each cluster, Seurat’s FindAllMarkers tool was used to conduct a differential gene expression analysis across cell clusters (Zhang et al., 2019; Huang et al., 2024; Ding et al., 2023). After that, we used the singleR package to identify and label various cell clusters according to the patterns of marker gene composition. These were then manually confirmed and improved using the CellMarker database.

### 2.4 Preference analysis of cells

Odds ratios were computed using the methods outlined in the study to evaluate the preference of tumor cell subtypes for cancer (Zheng et al., 2021).

### 2.5 Cell stemness analysis

The authors used AUCell (Aibar et al., 2017), a technique for locating active genes in scRNA-seq data, to evaluate the stemness of TC subtypes. Furthermore, cell stemness was assessed using the CytoTRACE R package (version 0.3.3), which allowed for speculative inference about the chronological order of cell differentiation (Lin et al., 2024b; Sun et al., 2024; Li et al., 2024).

### 2.6 Trajectory analysis of TCs subtypes

The Monocle 2 algorithm (Qiu et al., 2017; Zhao et al., 2023; Feng et al., 2025), which condensed high-dimensional gene expression data into a lower-dimensional space for display, was used to predict pseudotime trajectories of TCs subtypes. Trajectories were created from the cells, and each branch point was recognized. The Slingshot R package (version 2.6.0) was used to investigate lineage and pseudotime links in more detail (Zhou et al., 2024; Zhao et al., 2024b; Shao et al., 2024). This technique fitted branching trajectories with synchronized master curves and created lineage structures using clustering-based minimal spanning trees.

### 2.7 Analysis of cellular subtype enrichment

Using the ClusterProfiler R package (version 4.6.0), we enriched differentially expressed genes (DEGs) using Gene Ontology (GO) (Zhao et al., 2022a; Lin et al., 2022b; Zhao Z. J. et al., 2021), Kyoto Encyclopedia of Genes and Genomes (KEGG) (Wang et al., 2020; Zhao Z. et al., 2021), and Gene Set Enrichment Analysis (GSEA) (Zhang et al., 2023b; Chen Y. et al., 2022a; Sun et al., 2022a). We set significance of all terms at an adjusted p-value whose threshold was less than 0.05 and analyzed the data (Alexa et al., 2006). The filtering criteria for fold change were based on a log2 fold change threshold of 1, which means that only genes with a fold change greater than or equal to 1 (or less than or equal to −1) were considered as differentially expressed.

### 2.8 Gene regulatory network analysis

SCENIC identified active transcriptional regulators from single-cell data. The pySCENIC R software package (version 0.10.0) and Python (version 3.7) for single-cell regulatory network inference and clustering analysis were utilized to identify transcription factors (TFs) with notable expression differences in various TCs subtypes. To start, we used GRNBoost to find putative target genes linked to every TF. In order to find potential sites for direct binding, more DNA-motif analysis was carried out. AUCell has been used to evaluate the activity of the regulators in individual cells, and the top five TFs have been chosen based on the results. The website https://reorigins.aertslab.org/cistarget/ is where the human gene-motif rankings were first created. The Connection Specificity Index (CSI) methodology has been used to define regulatory modules in order to find certain association partners (Suo et al., 2018). Lastly, we classified different regulator modules using hierarchical clustering based on Euclidean distance. Studying the relationships between various regulators is made easier by building the regulator linkage network with a threshold value of 0.65.

### 2.9 Molecular signaling communication analysis

CellChat inferred ligand-receptor-mediated intercellular communication. Depending on the ligand-receptor level, regulatory frameworks were constructed and interactions were analyzed using the CellChat R software program (version 1.6.1) (Jin et al., 2021; Lin et al., 2024c; Liu et al., 2024). Using the “netVisualDiffInteraction” and “IdentifyCommunicationPatterns” features, we estimated the number of communication patterns and visualized variations in intercellular communication strength, setting a significant level where the p-value was 0.05.

### 2.10 Spatial transcriptomics deconvolution and analysis of cellular interactions

We utilized the integrated scRNA-seq dataset as a reference to execute cell type decomposition within the histological structures of the ST slide, employing the robust cell type decomposition (RCTD) approach with doublet_mode set to “full” (Cable et al., 2022).

Cell-cell interactions were assessed using stLearn, followed by a modified pseudo-time trajectory analysis in spatial contexts *via* stLearn (Pham et al., 2023), which utilized PAGA trajectory analysis based on tissue-wide SME normalized gene expression data to reveal relationships among subtypes. A pseudotime spatial trajectory algorithm was utilized to describe the malignant growth across the sections, identifying spatial and transcriptional connections among the subtypes.

### 2.11 Building prognostic models

We investigated the impact of ovarian cancer-associated TCs on patient survival prediction using key marker genes unique to crucial TCs subtypes. To find the most significant predictive genes, LASSO regression (Zheng R. et al., 2022; Zheng R. Z. et al., 2022; Li X. Y. et al., 2022) was performed after univariate Cox analysis. A risk score model, which is defined as Risk score = $\sum_{i}^{n}Xi\times Yi$ , was then established by doing multivariate Cox regression analysis to determine risk coefficients for each gene (Chen B. et al., 2022; Zou et al., 2022; Zhao J. et al., 2022). Using the “surv_cutpoint” tool to find an ideal threshold, patients were divided into low-risk and high-risk categories. The “Survival” R package (version 3.3.1) was used to analyze the survival of these cohorts (Lin et al., 2022c; Lin et al., 2022d; Lin et al., 2023), and the “ggsurvplot” function was used to show the survival curves. ROC curves were produced using the “timeROC” package (version 0.4.0) in order to evaluate the model’s prediction accuracy (Wang J. et al., 2022). AUC was then defined as the area under the ROC curve and responds to the accuracy of this predictive model (Zhao et al., 2022c). In order to purify transcriptomes from bulk data without distinguishing individual cells, CIBERSORT was also used to estimate the number of cell types in bulk RNA-seq data (Newman et al., 2019). Transcripts per million (TPM) values were used to pre-normalize the TCGA bulk RNA-seq data. Using 1,000 permutations without batch correction, a signature matrix was constructed using TPM-normalized datasets for particular cell types. To enable CIBERSORT to estimate the cell type fractions in both cohorts, ovarian cancer patients from the TCGA database were randomly assigned to training and testing cohorts in a 1:1 ratio according to survival status.

### 2.12 Analysis of immune infiltration

The CIBERSORT R program (version 0.1.0) was used to quantify immune cell infiltration and expression differences between various risk score groups. Correlations between immune cells and risk scores, modeled genes, and OS were then examined. Tumor purity, immunological score, EATIMATE score, and stromal score levels across different risk groups were assessed using the Xcell algorithm and the ESTIMATE R package (version 1.0.13).

### 2.13 Culture of cell lines

We used the American Type Culture Collection’s Caov-3 and SK-OV-3 cell lines, which we cultivated at 37°C, 5% CO_2_, and 95% humidity. The SK-OV-3 cell line was kept in MD10 medium with the same serum and antibiotic concentrations, whereas the Caov-3 cell line was cultivated in MD02 medium with 10% fetal bovine serum and 1% penicillin-streptomycin.

### 2.14 Transfection of cells

For every transfection, we utilized Lipofectamine 3000 RNAiMAX (Invitrogen, United States). Six-well plates were seeded with cells at 50% confluence, and the cells were transfected with the knockdown models (Si-FN1-1 and Si-FN1-2) and negative control (si-NC), respectively. GenePharma’s (Suzhou, China) short interfering RNA (siRNA) constructs were used to knock down FN1.

### 2.15 Test of colony formation

The cells were cultivated for 10 days after being seeded into six-well plates. The cells were then fixed for 15 minutes using 4% paraformaldehyde. The cells were then stained for 15 min using 0.5% crystal violet. Lastly, we used ImageJ software (National Institutes of Health, Bethesda, Maryland, United States) to take pictures of the colonies and determine their number.

### 2.16 Test of cell activity test

Transfected Caov-3 and SK-OV-3 cell lines were seeded at 5 × 10^3^ per well in 96-well plates, and their cell activity was assessed using the CCK-8 test. The cells were then cultivated for the entire day. We next filled each well with 10 μL of CCK-8 reagent (Vazyme, A311-01) and incubated them for 2 hours at 37°C in the dark. Lastly, on days 1, 2, 3, and 4 after transfection, we measured absorbance at 450 nm using a microplate reader (Thermo, A33978) and plotted the average OD values.

### 2.17 Test of transwell

Prior to the experiment, we first starved the cells for the entire day in serum-free media. The cell suspension was then added to the superior chamber of Costar plates that had been pre-treated with Matrigel (BD Biosciences, United States), while serum-enriched media was present in the inferior chamber. Following 2 days of incubation, we treated the cells with 4% paraformaldehyde and evaluated their capacity for invasion using crystal violet staining.

### 2.18 Test of wound-healing

Stable transfected cells were put into 6-well plates, and their growth was monitored until confluence. We made a scratch in each well using a sterile 200-μL pipette tip, cleaned the wells with PBS to get rid of any debris, and then incubated them in a medium free of serum. We took pictures of the scratch both then and 48 h later, and we measured its width using ImageJ software.

### 2.19 Proliferation test of 5-Ethynyl-2′-deoxyuridine (EdU)

In 6-well plates, 5 × 10^3^ transfected Caov-3 and SK-OV-3 cells were added to each well, and the cells were cultivated for the entire night. In the meanwhile, we combined 10 mM EdU with serum-free medium to create a 2× EdU working solution. Following a 2-h incubation period at 37°C, we rinsed the cells with PBS, fixed them for 30 min with 4% paraformaldehyde, and permeabilized them for 15 min with 2 mg/mL glycine and 0.5% Triton X-100. After that, we dyed them for half an hour at room temperature using 1X Apollo and 1X Hoechst 33,342. Finally, cell proliferation was evaluated using fluorescence microscopy.

## 3 Results

### 3.1 ScRNA-seq single-cell mapping found six primary cell types in ovarian cancer

**FIGURE 1:**
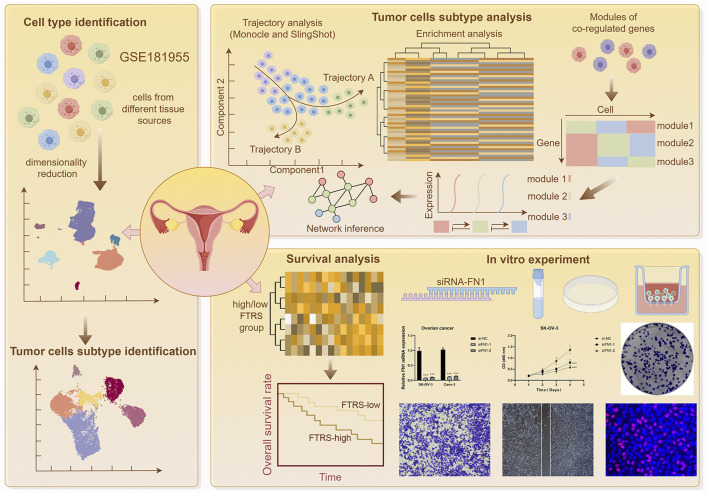
Summary chart. The concept and methodology of our entire paper were illustrated in the graphic. Prior to clustering and evaluating the TCs, we first acquired the raw data of ovarian cancer patients from GEO. Next, we clustered the main cells within the data. Second, we performed a number of analyses for the TCs, such as communication, transcription factor, enrichment, and trajectory analyses. We then identified the relevant subtype and developed a predictive model using the subtype. Lastly, using the signaling molecules of interest identified by the communication analysis, we conducted *in vitro* studies.

**FIGURE 2:**
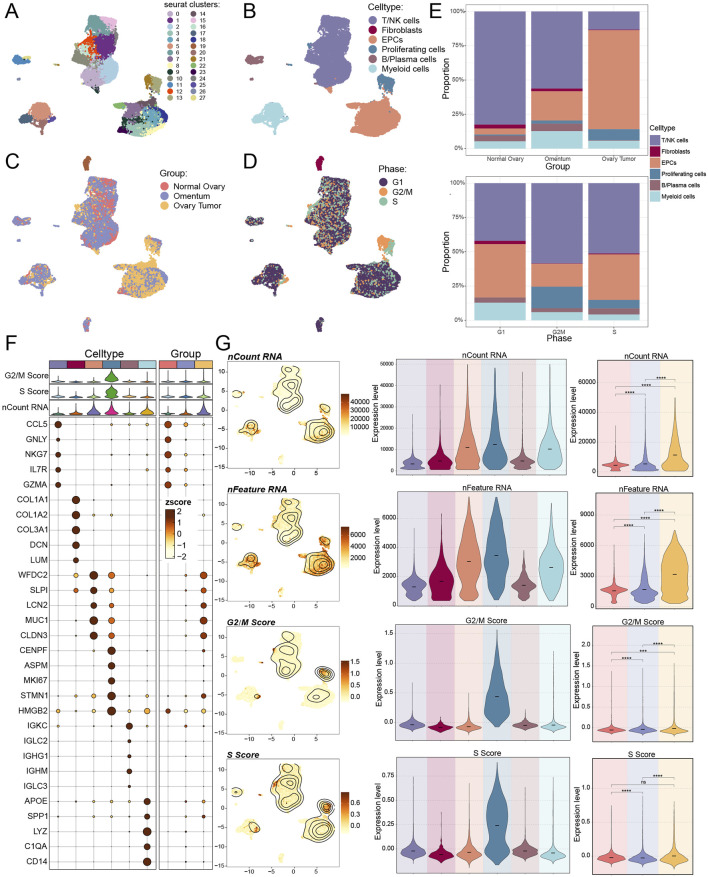
Single-cell mapping of ovarian cancer. **(A–D)** The UMAP plot **(A)** displayed the distribution of 28 cell clusters in both normal participants and ovarian cancer patients; six primary cell types were subsequently clustered **(B)**. Additionally, the UMAP figure displayed the distribution of tissue origins **(C)** and cell phases **(D)**. **(E)** The proportions of various cell types from different tissue origins (above) and cell phases (below) were displayed by bar graphs. **(F)** Using an enrichment bubble plot, the top five marker genes for ovarian cancer were shown to have differential expression across the six main cell types and three tissue sources. Bubble colors were associated with zscore, or normalized data. **(G)** UMAP plots and violin plots, respectively, were used to show the expression levels of nCount-RNA, nFeature-RNA, G2/M.Score, and S. Score in each cell type and tissue origin. And ns represented difference not significant, *** represented p < 0.001, **** represented p < 0.0001.

Initially, eight tissue samples—normal ovarian tissue, omental tissue, and ovarian cancer tissue—were obtained from GEO to ascertain the cell types implicated in the progression of ovarian cancer. We would conduct a series of multi-omics analyses and experimental validation of the obtained sample data (Figure 1). Genes failing to meet the minimal expression threshold were excluded following an assessment of the quality and completeness of the raw data. Following the removal of batch effects and quality control, 35,726 cells were retained and categorized into 28 clusters (Figure 2A). Through the analysis of cellular genetic profiles and prevalent markers, these 28 cell clusters were conclusively identified as six principal cell types: fibroblasts (*DCN*), myeloid cells (*LYZ*), proliferating cells (*MKI67*), epithelial cells (EPCs, *WFDC2*), T/NK cells (*CCL5*), B and plasma cells (*IGKC*) (Figure 2B). The fraction of EPCs was increasing, but the proportion of T/NK cells was decreasing in ovarian cancer tissues, omental tissues, and normal ovarian tissues. Specifically, myeloid cells exhibited a decline from omental tissue to ovarian cancer tissue and an increase correspondingly from normal ovarian tissue to omental tissue. EPCs predominantly resided in the G1 and S phases, whereas T/NK cells were more prevalent in the G1, G2/M, and S phases, with G2/M being the most frequent (Figures 2C–E). The main enriched genes in the bubble diagram for each cell type corresponded with the known marker genes (Figure 2F). Finally, the quantity of RNAs expressed in EPCs was illustrated by violin plots and UMAP plots, which further indicated that the characteristic RNAs were predominant across all cell types (Figure 2G). Ovarian cancer tissues demonstrated significantly elevated expression levels of both items in comparison to other tissues. We additionally associated EPCs with ovarian cancer, as prior studies indicated that ovarian cancer originates from three distinct tissue types: around 3 to 5 percent from germ cells, 5 to 8 percent from stromal cells, and 85 to 95 percent from epithelial cells.

### 3.2 Utilizing single cells for the purpose of analyzing tumor cells in ovarian cancer

**FIGURE 3:**
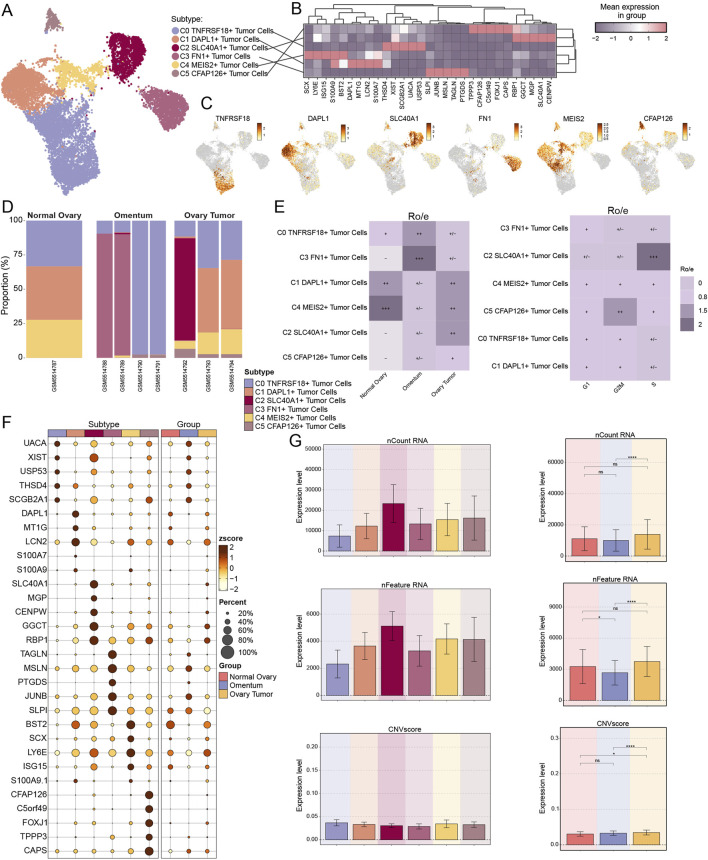
Single cell analysis in tumor cells (TCs). **(A)** six subtypes of TCs from ovarian cancer patients were displayed by UMAP plot. **(B)** The first five marker genes for different TCs subtypes were shown by the heatmap, with purple representing high expression. **(C)** The expression and distribution of the six TCs subtypes named genes were demonstrated by UMAP plot. **(D)** The proportion of TCs subtypes from each sample origin was displayed by bar graphs. **(E)** Ro/e score was used to estimate tissue origins (left) and cell phases (right) preference of different TCs subtypes. **(F)** Differential expression of the first five marker genes in the 6 TCs subtypes and in the three tissue origins were shown by bubble plot. Bubble colors are linked to normalised data (zscore). **(G)** Bar graphs displayed the expression levels of nCount-RNA, nFeature-RNA and CNVscore in each TCs subtype as well as tissue origin. And ns represents difference not significant, * represented p < 0.05, **** represented p < 0.0001.

We then utilized inferCNV to distinguish malignant TCs from EPCs due to the robust correlation between EPCs and ovarian cancer TCs (Supplementary Figure S1). We chose T cells and NK cells as reference cell types for the inferred CNV analysis based on their well-established role as non-malignant, genetically stable immune populations within the tumor microenvironment. These cells are not expected to harbor tumor-specific genomic alterations, making them appropriate baselines for distinguishing CNV signals that are characteristic of malignant cells. To distinguish various TCs subtypes, we down-clustered the 10,953 TCs identified according to the flag genes produced by the cells. The subtypes were subsequently assigned their corresponding genes: C0 *TNFRSF18*+ TCs, C1 *DAPL1*+ TCs, C2 *SLC40A1*+ TCs, C3 *FN1*+ TCs, C4 *MEIS2*+ TCs, and C5 *CFAP126*+ TCs (Figures 3A–C). C0 *TNFRSF18*+ TCs were found in all three tissues, C1 *DAPL1*+ TCs and C4 *MEIS2*+ TCs were more common in both normal and tumor tissues overall, and C2 *SLC40A1*+ TCs were most common in one of the tumor samples. The analysis found that the omental tissues were dominated by C3 *FN1*+ TCs (Figures 3D,E). The propensity of ovarian cancer cells to disseminate to the omentum and the incidence of intraperitoneal metastases are well recognized (Arie et al., 2013; Miyamoto et al., 2023). Furthermore, the omentum was among the initial sites where malignancies establish or disseminate into the peritoneal cavity, serving as a vital pre-metastatic environment for the progression of invasive disease (Etzerodt et al., 2020; Bella et al., 2022). A comparable study indicated that *FN1*, the gene associated with C3 *FN1*+ TCs, served as a marker correlated with unfavorable outcomes, while another investigation revealed its involvement in regulating the advancement of ovarian cancer (Wei et al., 2021; Meagher et al., 2022). The precise mechanism by which it led to ovarian cancer remained unidentified. The amplification of *JUNB* was associated with a worse outcome in ovarian cancer patients, while the gene *MSLN*, prevalent in C3 *FN1*+ TCs, was previously thought to have a role in the peritoneal dissemination of ovarian TCs (Hilliard, 2018; Perez-Benavente et al., 2022) (Figure 3F). These investigations further corroborated our previous hypotheses. Consequently, ovarian tumor cells were likely to establish the initial cluster of tumors in the omentum, which would then generate malignant cancer cells and ultimately result in ovarian cancer. Furthermore, we might deduce that C5 *CFAP126*+ TCs predominantly favored the G2/M phase, while C2 *SLC40A1*+ TCs exhibited a high preference for the S phase. We subsequently calculated the variances in nCount RNA, nFeature RNA, and CNV score expression levels among subtypes and tissue sources (Figure 3G). It would be beneficial for us to examine the pathways of the TCs from omental tissues to ovarian cancer tissues, as the three results exhibited notable discrepancies between ovarian cancer and omental tissues, highlighting significant heterogeneity between these two tissue types.

### 3.3 Cellular stemness of CytoTRACE AUC score showed C3 FN1+ TCs hyperdifferentiation with high cellular stemness

**FIGURE 4:**
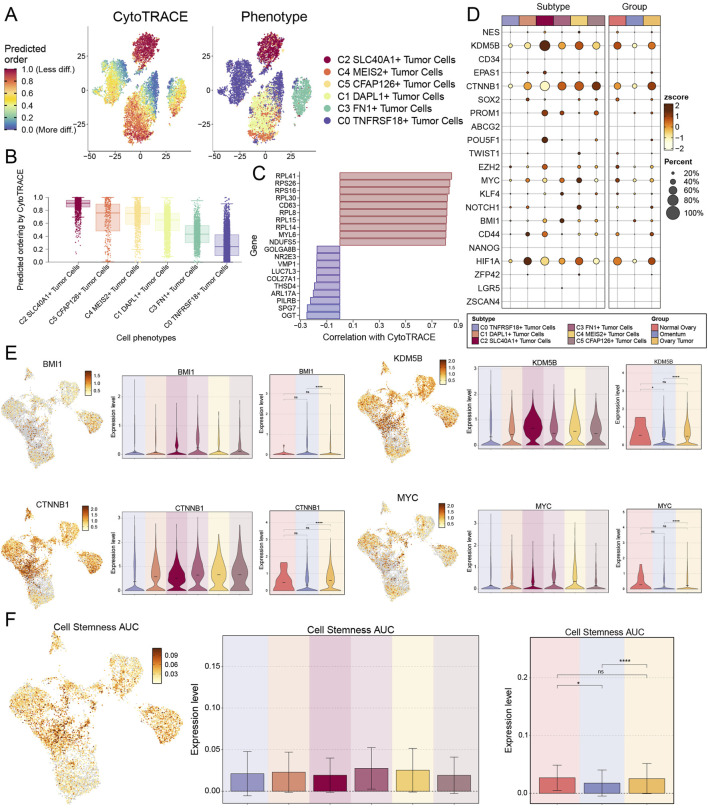
Cell stemness analysis of TCs subtypes. **(A)** The distribution of CytoTRACE values for TCs subtypes was shown by the left panel. Colors represented upper or lower cell stemness. And the spatial distribution of TCs subtypes was shown by the right panel. **(B)** The TCs subtypes were arranged based on the CytoTRACE prediction order and presented in boxline plots. **(C)** The genes associated with CytoTRACE were showed in bar graph, where greater than 0 is a positive association shown in red and less than 0 is a negative association shown in blue. **(D)** Bubble plot showed the expression levels of stemness genes in each TCs subtype and tissue origin. **(E)** Four stemness genes (BMI1, CTNNB1, KDM5B and MYC) expressed in each TCs subtype and tissue origin were presented in UMAP and violin plots. And ns represents difference not significant, * represented p < 0.05, **** represented p < 0.0001. **(F)** UMAP plot and bar graphs showed the AUC values and their distribution for cell stemness for each TCs subtype and tissue origin. And ns represents difference not significant, * represented p < 0.05, **** represented p < 0.0001.

Subsequently, to assess the malignancy level of the TCs in each subtype, we utilized CytoTRACE to gain an early insight into the differentiation extent of each subtype (Figures 4A–C). C2 *SLC40A1*+ TCs and C5 *CFAP126*+ TCs, characterized by a significant prevalence in ovarian cancer tissues, demonstrated a low degree of differentiation, suggesting a higher malignancy in these two subtypes of TCs. This was consistent with the tissue percentage and tissue preference of each subtype found in Figures 3D,E. We focused on C3 *FN1*+ TCs, which exhibited a relatively lower degree of malignancy, suggesting that they might still be in the growth and development phase rather than fully mature. This corroborated our previous hypothesis that malignant TCs linked to ovarian cancer may develop in the omentum. Bubble plots, which were displayed as violin plots and UMAP plots, showed that the stemness genes enriched to C3 *FN1*+ TCs were primarily *BMI1, CTNNB1, KDM5B*, and *MYC* (Figures 4D,E). Significant differences in expression levels were observed for these four genes between ovarian cancer and omental tissues. Expression levels were markedly elevated in ovarian cancer tissues compared to omental tissues, suggesting a potential role in facilitating the malignant proliferation of TCs within omental tissues. Previous studies have demonstrated that BMI1 promotes TCs growth and metastasis in ovarian cancer by altering TCs angiogenesis and extracellular matrix structure, primarily through the regulation of adhesion plaques and the *PI3K/AKT* signaling pathway (Zhao et al., 2018). The *Wnt/β-catenin* pathway, established by *CTNNB1* encoding β-catenin, was a crucial signaling pathway implicated in the epithelial-mesenchymal transition (EMT) and has been shown to play a significant role in the carcinogenesis of ovarian cancer. (Arend et al., 2013). Ultimately, we evaluated the stemness of each subtype’s cells using AUC scoring, revealing that C3 *FN1*+ TCs obtained a high score, indicating their potential to promote the proliferation of malignant TCs (Figure 4F).

### 3.4 C3 FN1+ TCs were found at an early stage of tumor formation

**FIGURE 5:**
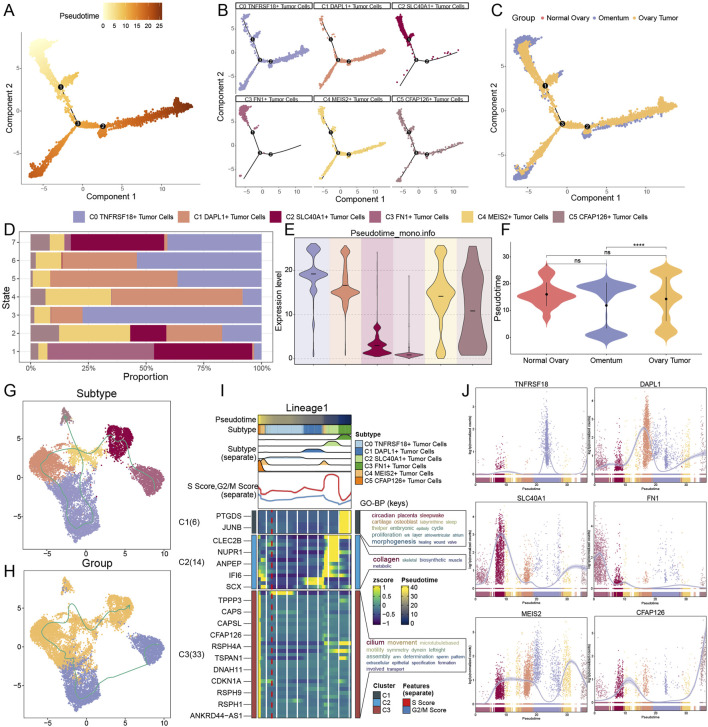
Prediction of developmental differentiation trajectories in TCs. **(A–C)** Monocle 2-predicted trajectories of TCs differentiation were displayed by pseudotime trajectory plots, which showed the distribution of Monocle-predicted pseudotemporal order **(A)**, subtypes **(B)** and tissue origins **(C)**, respectively. **(D)** Considering the proportion of TCs subtypes at each of the seven time points, the order was ranked according to the pseudotemporal state. **(E and F)** Pseudotime order expression of each TCs subtype **(E)** and tissue origin **(F)** was shown by violin plots, with lower expression levels representing a more advanced order of expression. And ns represents difference not significant, **** represented p < 0.0001. **(G and H)** Differentiation trajectories’ distribution of six TCs subtypes **(G)** and tissue origins **(H)** simulated with the pseudotemporal order in TCs. **(I)** Heatmap showed the correlation features of the pseudotime trajectory system of TCs. Pseudotime values are linked to differentiation, with 0 indicating the beginning point and 40 indicating the ending point. **(J)** The trajectories of the named genes for the six TCs subtypes, showing changes along the lineage, were demonstrated by the dynamic trends plots obtained after SlingShot visualization.

We employed the Monocle and SlingShot methodologies for trajectory inference to examine the sequence of developmental trajectories of TCs subtypes. We initially constructed a graph depicting the differentiation trajectory of pseudotime utilizing Monocle (Figure 5A). C0 *TNFRSF18*+ TCs, C4 *MEIS2*+ TCs, and C5 *CFAP126*+ TCs were distributed at all developmental stages, whereas C1 *DAPL1*+ TCs was primarily disseminated in the latter stages of development, according to a comparison of the subtypes’ differentiation trajectories using Figure 5B. On the other hand, C3 *FN1*+ TCs essentially did not go beyond node 1, while C2 *SLC40A1*+ TCs and C3 *FN1*+ TCs were primarily seen in the early phases of tumor development. Figure 5C illustrated the differentiation trajectories from various tissue origins. The differentiation trajectory of TCs was divided into seven phases, with findings indicating that the highest proportion of C3 *FN1*+ TCs, the earliest subtype in development, was observed in stage 1 (Figure 5D). The violin plots illustrating the developmental sequence indicated that omental tissues developed prior to ovarian cancer tissues, exhibiting a statistically significant difference, with C3 *FN1*+ TCs identified as the earliest subtype of TCs (Figures 5E,F).

To further elucidate trajectories among all TC subtypes and tissues, we subsequently employed SlingShot analysis. As the diverse tissues advanced in the sequence of omental-ovarian cancer, with minimal involvement from normal tissues in the developmental process, the distinct subtypes evolved and differentiated in the subsequent order: C3 *FN1*+ TCs, C2 *SLC40A1*+ TCs, C1 *DAPL1*+ TCs, C0 *TNFRSF18*+ TCs, C4 *MEIS2*+ TCs, and C5 *CFAP126*+ TCs (Figures 5G,H). The results of SlingShot’s analysis aligned with Monocle’s findings, indicating that omental tissue and C3 *FN1*+ TCs subtypes were likely origins of ovarian cancer formation. Examining the role of C3 *FN1*+ TCs in the malignant proliferation of TCs within omental tissue, leading to carcinogenesis, might provide new insights for targeted therapies in ovarian cancer. The functional processes associated with the lineage trajectory of TCs subtypes were subsequently determined using GO-BP enrichment analyses (Figure 5I). Lastly, the dynamic trend diagrams were used to illustrate the distribution and variations in expression of specific genes across TCs subtypes in pseudotime (Figure 5J). The initial high expression of *FN1*, the gene that wass named for C3 *FN1*+ TCs, and the subsequent low level of fluctuation state following an abrupt decline piqued our interest. This illustrated the importance of C3 *FN1*+ TCs being highly active during the initial stages.

### 3.5 The C3 FN1+ TCs subtype promoted the development of malignant TCs through various mechanisms

**FIGURE 6:**
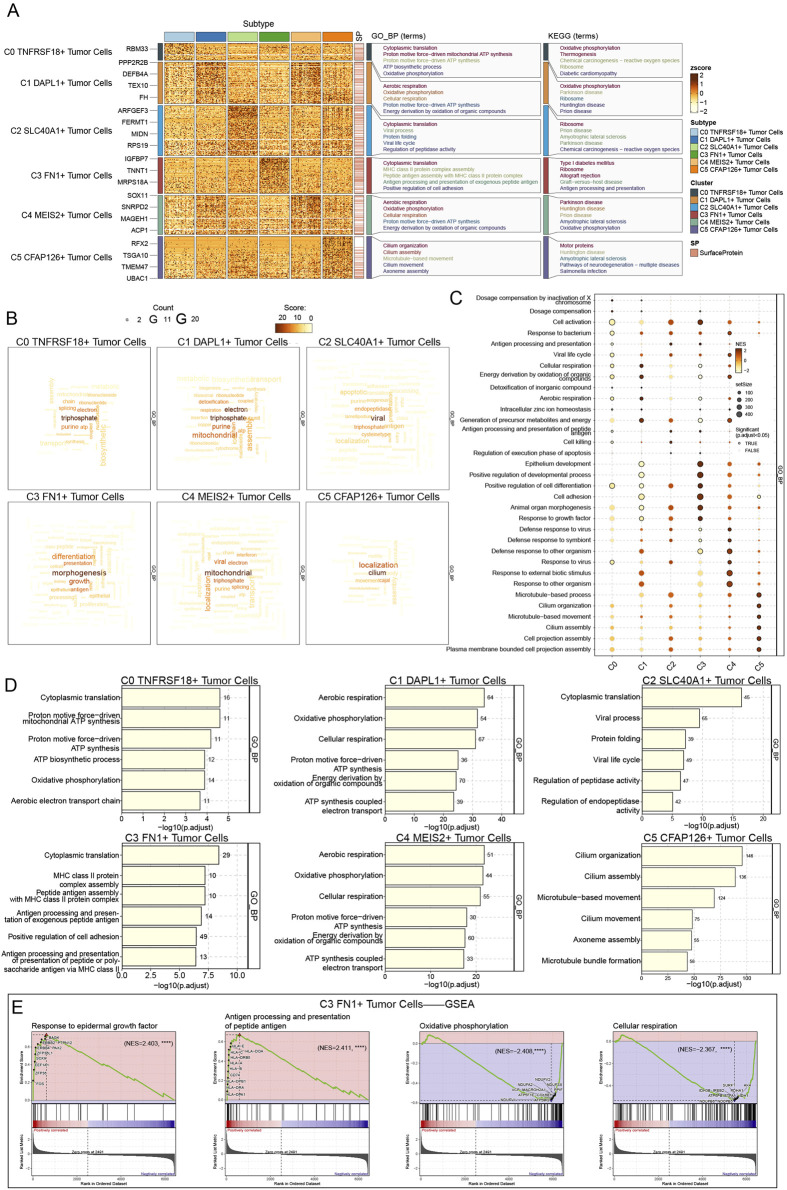
Enrichment analysis of TCs for genes and pathways. **(A)** The GO-BP and KEGG enrichment term scores were displayed by heatmap. **(B)** The activity of different pathways in TCs subtypes was demonstrated by the word cloud diagrams. **(C)** GSEA analysis map depicting various pathways in each TCs subtype was generated. **(D)** The bar graphs revealed the GO-BP results for each TCs subtype separately. **(E)** GSEA results among C3 FN1+ TCs.

We performed enrichment pathway analyses to examine the role of C3 *FN1*+ TCs in tumor regeneration and to understand the specific functional activities of these cells during early developmental stages. It was intriguing to observe that the pathway identified by GO-BP enrichment analysis of C3 *FN1*+ TCs was cytoplasmic translation, a mechanism that was closely associated with TCs proliferation (Figures 6A,D). Furthermore, prior research has shown that protein translation promoted the growth of ovarian cancer tumors, a mechanism that was similar to the one we have identified (Guo et al., 2019). Moreover, the KEGG enrichment analysis was used to identify the critical pathways for C3 *FN1*+ TCs, including ribosome, allograft rejection, and type I diabetes mellitus. It was intriguing to observe that we collected research that established type I diabetes mellitus as a risk factor for cancer and that individuals with this condition were more likely to develop ovarian cancer (Swerdlow et al., 2023; Suh and Kim, 2019). Utilizing the word cloud, we identified morphogenesis as the most relevant pathway associated with C3 *FN1*+ TCs, indicating its involvement in the early development of ovarian cancer (Figure 6B). The hypothesis was validated through a review of relevant literature, which revealed that multiple genes associated with tissue and cellular morphogenesis play a role in the development of ovarian cancer (Shi-Peng et al., 2017; Chen et al., 2021). GSEA has identified enriched activities including the development of epithelium, cell differentiation, positive regulation of developmental processes, cell adhesion, morphogenesis of animal organs, and growth factor response (Figure 6C). The cancer cells from the omentum spread and adhered to the ovary, leading to the development of ovarian carcinoma and morphological changes in the ovary. Additionally, the epithelial cells in C3 *FN1*+ TCs experienced mutations during development to form TCs, which subsequently responded to growth factors, proliferated, and differentiated, ultimately resulting in the formation of malignant proliferating cells. Finally, we identified upregulated and downregulated pathways in C3 FN1+ TCs through GSEA once again (Figure 6E). The pathway was enhanced in response to antigen processing, peptide antigen presentation, and epidermal growth factor. Epidermal growth factor has been demonstrated to enhance ovarian epithelial cell proliferation, facilitate EMT, elevate cancer cell invasiveness and drug resistance, and negatively impact patient differentiation and prognosis (Rodriguez et al., 1991; Grassi et al., 2017; Oh et al., 2014).

### 3.6 The role of C3 FN1+ TCs subtypes in the development of ovarian cancer through the M2 regulatory factor module

**FIGURE 7:**
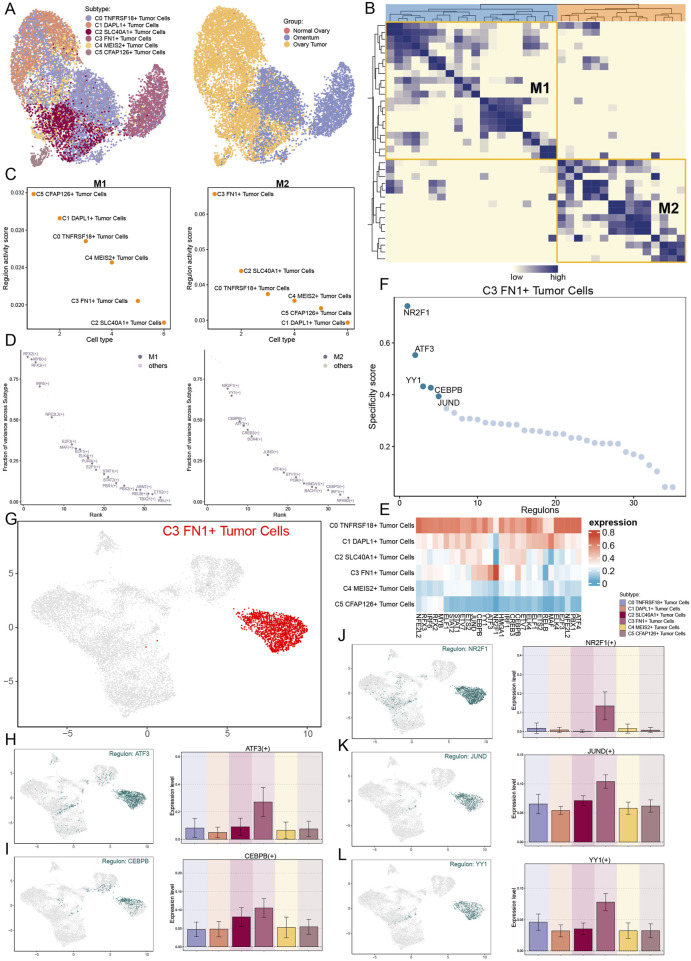
Analysis of upstream transcript levels for TCs. **(A)** UMAP plots of all TCs were visualised according to regulator activity. **(B)** Based on regulon connection specificity index matrix, regulon modules were identified. **(C)** Regulon activity scores of different TCs subtypes in regulatory modules M1 and M2. **(D)** Expression ranking of regulatory modules M1 and M2 related genes in all TCs. **(E)** The heatmap illustrated the expression of the regulators in each TCs subtype, with red indicating higher expression and blue indicating lower expression. **(F and G)** Regulated genes highly expressed in the C3 FN1+ TCs subtype and their expression distribution in UMAP. **(H–L)** The expression of the five highly expressed genes (ATF3, CEBPB, NR2F1, JUND and YY1) in the C3 FN1+ TCs subtype and their distribution were shown separately.

We employed pyscenic to examine the gene regulatory architecture of C3 *FN1*+ TCs to understand the influence of TFs on cellular activities. A preliminary categorization of all TCs based on regulatory activity was conducted initially (Figure 7A). The UMAP plot derived from the activities of the TFs exhibited reduced discretization, as indicated by the clustering results, thereby excluding confounding variables. The C3 *FN1*+ TCs exhibited limited discretization and were predominantly located on the right side of the figure. Based on the CSI, we divided the TF subtypes into two regulatory factor modules, M1 and M2, in order to see the correlation between them (Figure 7B). Each of these two regulatory factor modules gathered TFs that might potentially work together to regulate genes. In the M2 regulatory module, we could observe that C3 *FN1*+ TCs had the greatest regulon activity score (Figure 7C). As a result, the primary regulators of the transcription carried out by C3 *FN1*+ TCs were also the regulatory factors grouped in M2. *ATF3, CEBPB, NR2F1, JUND*, and *YY1* were the five main regulators in C3 *FN1*+ TCs, as shown in Figures 7D–F. We demonstrated the distribution of these five regulators on UMAP and the expression of each in the various TCs subtypes (Figures 7G–L). Almost all five regulators exhibited low expression in other TCs subtypes while demonstrating high expression in C3 *FN1*+ TCs, suggesting a specialized expression pattern. Research indicated that cells from recurrent solid tumors demonstrated increased expression of *ATF3*, implying its role in promoting ovarian cancer aggressiveness, treatment resistance, and recurrence (Bopple et al., 2024). Furthermore, elevated *JUNB* levels have been shown to enhance tumor growth and metastasis in mice by altering the *TGF-β2*-stimulated response from an antiproliferative to a pro-invasive one. Additionally, tumor genomic data suggest that *JUNB* amplification correlates with poor prognosis in ovarian cancer patients (Perez-Benavente et al., 2022). The most notable finding was that *NR2F1*, absent in nearly all other subtypes, regulated the *TGF*-*β1*-driven EMT, influencing immunological response and platinum sensitivity (Liang et al., 2022). In a nutshell, TF analyses suggested that C3 *FN1*+ TCs played a promoting role in ovarian carcinogenesis and were associated with poor prognosis.

### 3.7 An examination of the global communication of cells diagnosed with ovarian cancer

**FIGURE 8:**
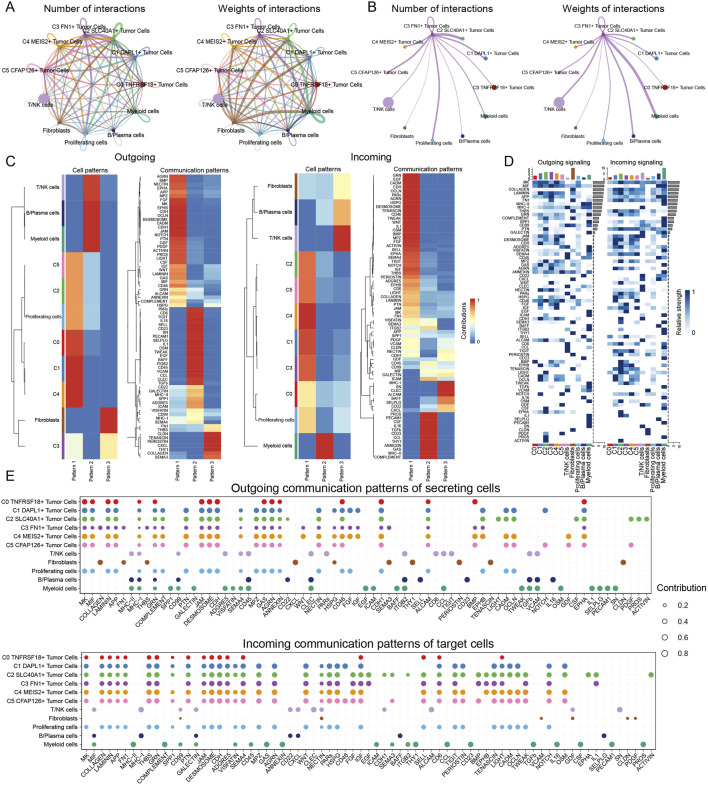
Analysis of communication interactions of various cells in ovarian cancer. **(A)** The count (left) and weights (right) of cell interactions in ovarian cancer were demonstrated by circle plots. **(B)** The C3 *FN1*+ TCs subtype was taken as the sender and its interactions with the remaining cells were analysed. **(C)** Heatmap demonstrated pattern recognition of all-cell interactions. The outgoing (left) and incoming (right) plots showed three pattern recognition cases for incoming signals and three pattern recognition cases for outgoing signals. **(D)** Ligands and receptors linked to the outgoing and incoming signals of cell interactions was displayed by heatmap. **(E)** The secretory cell communication patterns among various cells of ovarian cancer were demonstrated by outgoing and incoming contribution bubble plots.

For the purpose of defining and comprehending complex biological interactions, we conducted research on communication webs between ligands and receptors as well as intercellular linkages. Using CellChat analysis, we initially reported the intercellular communication webs that were present in ovarian cancer, which included a large number of cell types and a variety of TCs subtypes (Figure 8A). Concurrently, utilizing the C3 *FN1*+ TCs subtype of interest as the originating cell, we established a communication network for it (Figure 8B). We observed a notable cellular association between proliferating cells and myeloid cells, as well as C3 *FN1*+ TCs cells. To understand the communication patterns in ovarian cancer, we analyzed the relationship between the populations of cells that released and received signaling molecules and the fundamental communication dynamics among the cells (Figure 8C). This discovery enabled us to identify three distinct patterns of incoming and outgoing signaling, together with the signaling molecules associated with each pattern. Subsequently, we employed heatmaps to illustrate the expression of numerous signaling molecules in both incoming and outgoing signaling pathways across different cell types and TCs subtype, respectively (Figure 8D). The efferent signaling pathway in C3 *FN1*+ TCs exhibited substantial expression of *MIF*, *APP*, *FN1*, *AGRN*, *WNT*, and *NECTIN*. Finally, we measured the ligand-receptor network to ascertain the exact outgoing and incoming cellular communication patterns relevant to the six TCs subtypes (Figure 8E). C3 *FN1*+ TCs predominantly accepted *THBS*, *GRN*, and *EGF* signaling molecules in incoming communication, while they primarily emitted *COLLAGEN*, *FN1*, and *SEMA3* signaling molecules in outgoing communication. The focus of our investigation was the *FN1* dominant signaling pathway. Additional research was required to comprehensively elucidate the impact of *FN1* signaling molecules on ovarian cancer development.

### 3.8 Further examination of the pro-cancer role of the FN1 signaling pathway

**FIGURE 9:**
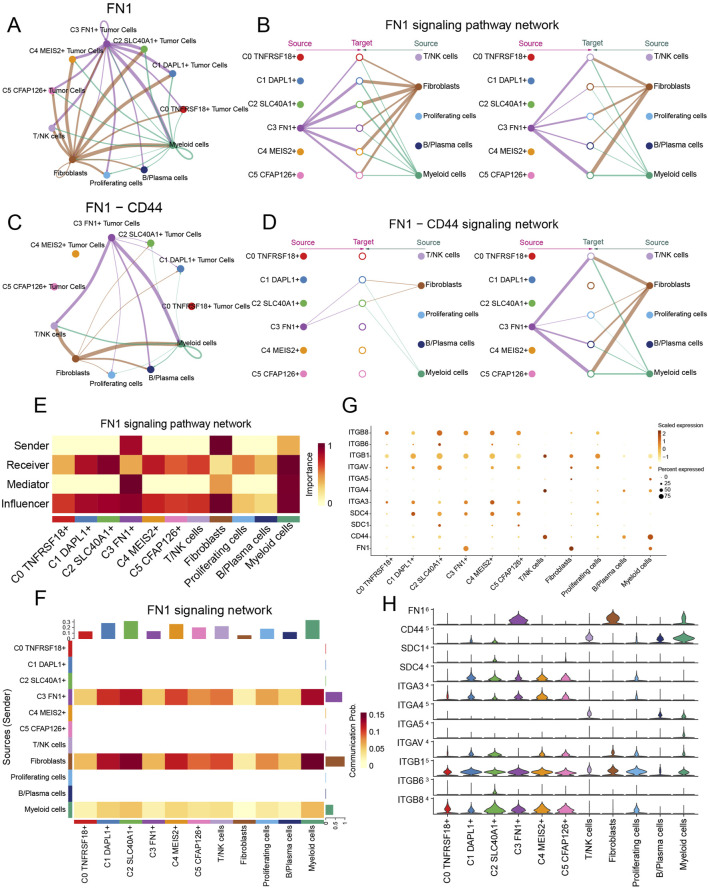
Visualization of the FN1 signaling pathway. **(A–D)** Circle and hierarchical diagrams depicted FN1 signaling **(A and B)** and the intercellular communication network of the FN1-CD44 signaling pathway **(C and D)**. **(E)** The centrality score of the FN1 signaling pathway was demonstrated by heatmap. **(F)** The cell interactions of the FN1 signaling pathway were demonstrated by heatmap. **(G and H)** Bubble plots **(G)** and violin plots **(H)** revealed cell interactions in the FN1 signaling pathways.

A circular diagram illustrated the intercellular communication framework, with *FN1* serving as the signaling molecule. A hierarchical diagram was utilized to refine the specific intercellular connections between cells utilizing *FN1* as the signaling molecule (Figures 9A,B). The findings indicated that C3 *FN1*+ TCs, fibroblasts, T/NK cells and myeloid cells exhibited significant signaling cross-talk, with *FN1* serving as the secreted ligand. Subsequently, we elucidated the intercellular communication network of the *FN1*-*CD44* signaling pathway and further identified the receptor *CD44* (Figures 9C,D). Myeloid cells and T/NK cells were two examples of ligand-receiving target cells associated with both targeted therapy and ovarian cancer. Myeloid cells, encompassing monocytes, granulocytes, dendritic cells, and macrophages, constituted a significant proportion of the TME in cancer and were crucial in regulating tumor spread (van Vlerken-Ysla et al., 2023). The molecular pathways facilitating interaction between macrophages and disseminated cancer cells might serve as innovative targets for the prevention of metastasis and disease recurrence. It was exhilarating to observe that tissue-resident macrophages in the omentum have been empirically demonstrated to facilitate the metastatic dissemination of ovarian cancer in prior studies (Etzerodt et al., 2020; An and Yang, 2021; Jazwinska et al., 2023). Furthermore, immunotherapy aimed at T cells in ovarian cancer suggested that the advancement of ovarian cancer was affected by the interactions between T cells and cancer cells (Blanc-Durand et al., 2023; Nasiri et al., 2023). Using the centrality measure method, which assessed the relative importance of each cell type in this process, the cell types mediating and influencing *FN1* signal-mediated intercellular communication were identified. Myeloid cells acted as receivers, mediators, and influencers within the *FN1* signaling pathway, whereas C3 *FN1*+ TCs were significantly expressed as senders, as illustrated in Figure 9E. The heatmap again demonstrated the significant signaling interactions generated by fibroblasts and C3 *FN1*+ TCs functioning as secretory cells on myeloid cells (Figure 9F). The findings demonstrated that C3 *FN1*+ TCs and fibroblasts primarily utilized *FN1* as a ligand to interact with myeloid cells, with *CD44* serving as the receptor. Bubble and violin plots depicted cell-cell interactions among several ligands, with *FN1* serving as the ligand (Figures 9G,H). These findings increased our interest in the study of *FN1* as well as C3 TCs subtype even more, so we next analyzed them further in the context of the clinic through the ST.

### 3.9 Spatial transcription analysis elucidated the spatial distribution of C3 FN1+ TCs subtypes

**FIGURE 10:**
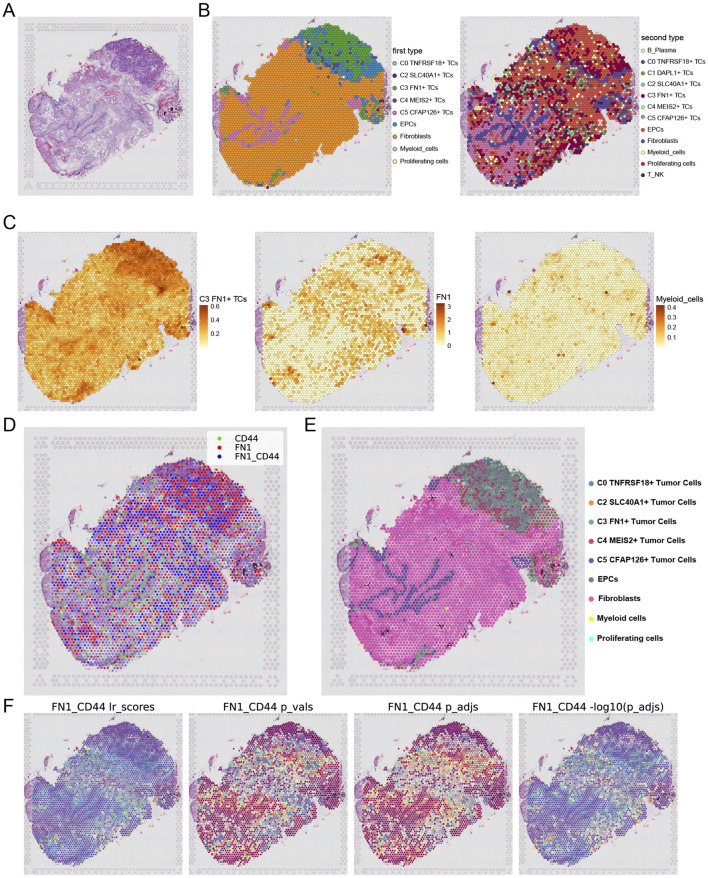
Analysis of spatial transcriptomics and cellular interactions. **(A)** Tissue section from a patient with ovarian cancer were shown. **(B)** Two results obtained by using RCTD for deconvolution. **(C)** The enrichment of the C3 and C5 subtypes as well as FN1 on the section was shown. **(D)** Spots were dichotomized as expressing ligand (red), expressing receptor (green), or both receptor and ligand (blue), with the criterion of whether the receptor expression was greater than the minimum expression threshold. **(E)** The direction indicated by the arrow indicated the FN1-CD44 cell interactions in section. **(F)** The statistical values of the FN1-CD44 interactions pairs in each spot were shown. lr_scores indicated the strength of the interactions in all spots and the p-values were shown. Darker colors indicated stronger communications.

We integrated scRNA-seq and spatial transcriptomics to elucidate the gene regulatory programs and cell-cell interactions that contribute to ovarian cancer development. Tissue sections were collected from an ovarian cancer patient, and ST data were processed (Figure 10A). We executed deconvolution with the RCTD approach to transfer cellular type labels from scRNA-seq data to ST data. Figure 10B presented the two examined outcomes. The C3 *FN1*+ TCs subtypes and EPCs were situated in the upper right quadrant of the slice, corresponding to the area identified as the tumor location in the tissue (Denisenko et al., 2024). Simultaneously, the locations of the C3 *FN1*+ TCs subtype and the C5 *CFAP126*+ TCs subtype corresponded to areas containing tumor tissue. Consequently, the data derived from ST indicated that the C3 *FN1*+ TCs subtype was indeed correlated with tumor formation in spatial locations. Subsequently, we illustrated the enrichment of spatial distributions for the C3 *FN1*+ TCs subtype, *FN1*, and myeloid cells. The findings indicated that the spatial locations of the C3 *FN1*+ TCs subtype and myeloid cells corresponded with the results derived from the RCTD, and that *FN1* was also present in the vicinity of the C3 *FN1*+ TCs subtypes (Figure 10C). We utilized stLearn for extrapolation to further examine the cellular interactions at specific spatial areas. Our prior research of *FN1*-*CD44* cell contacts in ovarian cancer demonstrated the expression of the receptor, ligand, and ligand-receptor in the corresponding sections (Figure 10D). The ligand *FN1* was mostly expressed by the C3 *FN1*+ TCs subtype and fibroblasts, with the area of elevated expression aligning with the spatial distribution of these two cell types. The expression region of the receptor *CD44* was situated between *FN1*, and there were areas within the tumor where both ligand-receptors were expressed. It was initially established that *FN1* and *CD44* interact through paracrine and proximal secretion mechanisms. Figure 10E illustrated a substantial quantity of cellular contacted in the area inhabited by fibroblasts, maybe linked to the preformation communication of the tumor. By evaluating the intensity of contacts across all areas in the tissue sections, we concluded that the intensity of cellular interactions was highest and statistically significant in the tumor region (Figure 10F). Our examination of the molecular and cytoarchitectural aspects of ovarian cancer corroborated the finding that the C3 *FN1*+ TCs subtype facilitated ovarian cancer *via* the *FN1*-*CD44* signaling pathway, and that they were linked through a paracrine cell-cell communication network inside the ovarian cancer milieu.

### 3.10 Create pertinent prognostic models to confirm clinical viability

**FIGURE 11:**
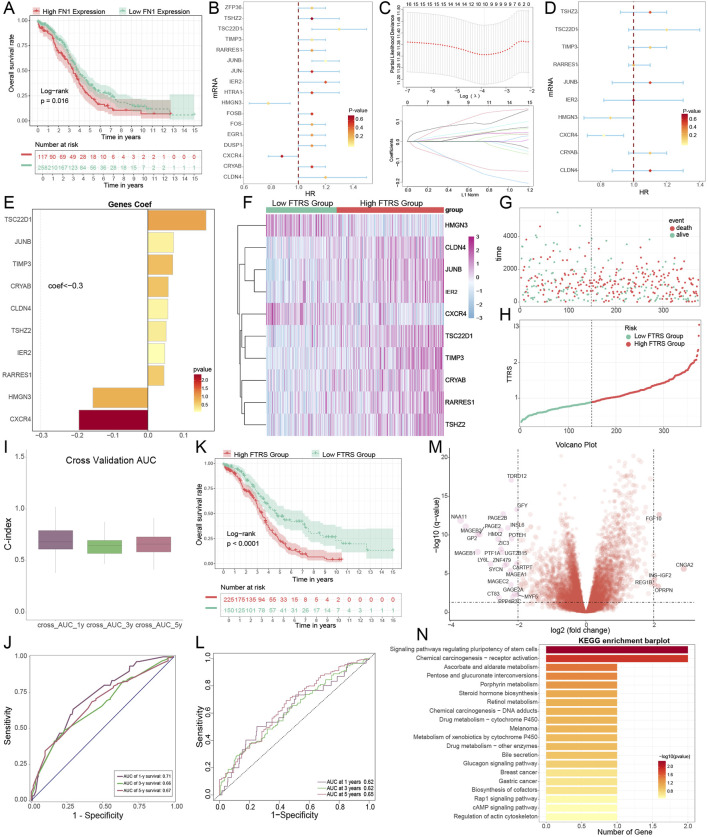
Construction of a risk score atlas for survival prognosis and enrichment pathway analysis in ovarian cancer. **(A)** Overall survival (OS) curves grouped by FN1 expression levels. **(B)** Forest plot of one-way cox regression analysis. **(C)** Values of the super-parameter λ were obtained by cross-validation using the minimum criterion in the LASSO-Cox model. The best lambda was used to generate a non-zero coefficient for the OS curve for the different scoring subgroups, where the optimal lambda yielded 10 non-zero coefficients. **(D)** Forest plot of multifactor Cox regression analysis. **(E)** The coefficient values of 10 mRNAs used for model building was showed by bar graph. **(F–H)** Risk profiles in the TCGA cohort. **(I)** The C-index of the AUC values for predicting 1-year, 3-year, and 5-year survival based on risk scores was depicted by the boxline plot. **(J)** The sensitivity and specificity of the risk scores for predicting 1-year, 3-year, and 5-year survival were depicted by the ROC curves. **(K)** OS curves for two scoring groups in a cohort. **(L)** The sensitivity and specificity of survival prediction for 1-year, 3-year, and 5-year survival were shown by the ROC curves. **(M)** Significantly DEGs were shown by the volcano plot. **(N)** The enrichment analysis results of differential genes in KEGG pathways for high and low FTRS groups were revealed by bar graphs.

The prognosis of patients classified according to strong or weak *FN1* expression was initially validated. The survival curves (Guan et al., 2023; Sun et al., 2022b) for the two groups exhibited significant differences, suggesting that *FN1* could serve as a valuable prognostic indicator for ovarian cancer (Figure 11A). Subsequently, univariate Cox regression analysis identified 17 mRNAs as potential predictive characteristics derived from the top 100 marker mRNAs of C3 *FN1*+ TCs (Figure 11B). Of these, it was discovered that *HMGN3* and *CXCR4* were protective factors (HR <1), whereas the other mRNAs were risk factors (HR >1). LASSO and multivariate regression analysis were employed to address multicollinearity among the mRNAs, ultimately identifying 10 genes associated with prognosis (Figures 11C,D). Analysis of the coefficient values of these prognostic mRNAs revealed that all were classified as risk mRNAs, except for *HMGN3* and *CXCR4* (Figure 11E). The study involved two groups: high and low FTRS (*FN1*+ TCs Risk Score) groups, aimed at investigating the influence of *FN1* high-expressing TCs on ovarian cancer patients, utilizing the 10 prognostic mRNAs identified from the TCGA cohort (Figures 11F–H). Furthermore, we confirmed that the model effectively predicted the OS C-index (Figure 11I). The model’s predictive accuracy was evidenced by the ROC curve (Figure 11J). The survival outcomes were notably poorer in the FTRS high-expression group, and there was significant variation in survival between groups categorized by FTRS (Figure 11K). We employed the ROC curve to evaluate the rigor of this prediction, and the findings demonstrated enhanced accuracy (Figure 11L). Furthermore, we illustrated the upregulation and downregulation of the initial 30 DEGs *via* volcano plots (Figure 11M). Subsequently, we examined the pertinent functional pathways with KEGG enrichment methodologies. The signaling pathways regulating stem cell pluripotency and receptor activation in chemical carcinogenesis were highlighted in KEGG (Figure 11N). Both pathways were associated with the initiation of ovarian carcinogenesis, corroborating our previous theories regarding the function of TCs that express *FN1*.

### 3.11 An investigation on the immunological infiltration that occurred in ovarian cancer

**FIGURE 12:**
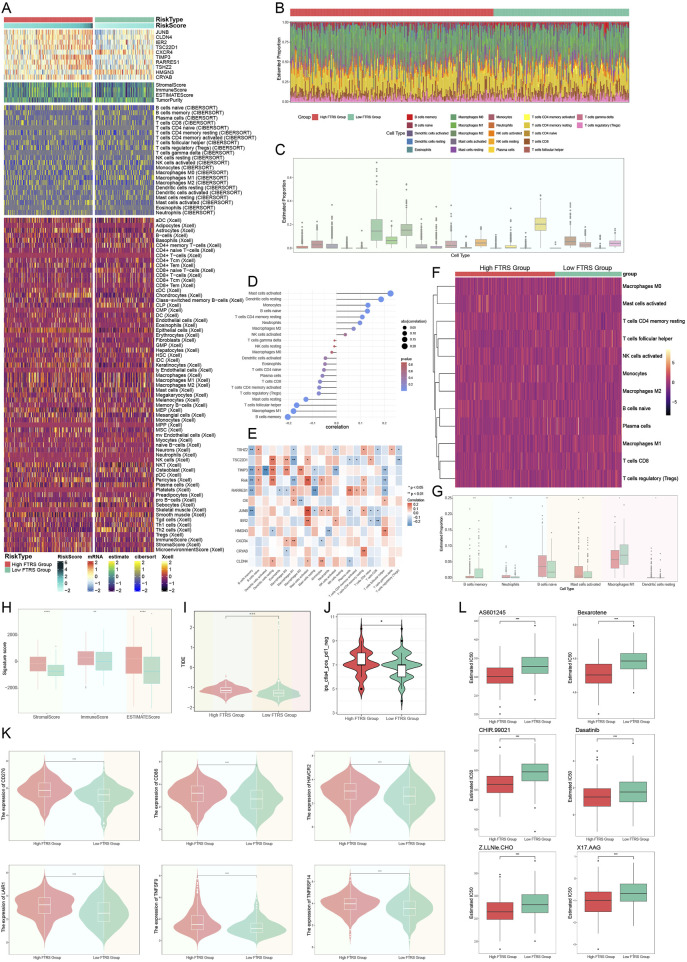
Analysis of immune infiltration in ovarian cancer. **(A)** The heatmap demonstrated the level of immune infiltration for different risk groups, which were analyzed using a variety of tools. **(B)** Immunoinfiltration analyses of different risk groups were performed with the CIBERSORT tool alone and visualized using heatmap. **(C)** The box line plot showed the predicted percentage of 22 immune cells in ovarian cancer. **(D)** The lollipop plot presented the correlation coefficients between the 22 immune cells and the risk score. **(E)** Heatmap of the correlation between 10 prognostic genes and 22 immune cells in ovarian cancer. And * represented p < 0.05, ** represented p < 0.01. **(F)** The heatmap further demonstrated the differences in the distribution of the selected 12 immune cells across the different risk groupings. **(G)** Box line plot demonstrated the differences in the distribution of the six immune cells in ovarian cancer among different risk groups, and all were statistically significant. And * represented p < 0.05, ** represented p < 0.01, *** represented p < 0.001. **(H)** Immune score, stromal score, and estimate score were computed for the high- and low-FTRS groups. And ** represented p < 0.01, **** represented p < 0.0001. **(I)** The difference in TIDE scores between the two risk subgroups was demonstrated by violin plot. And *** represented p < 0.001. **(J)** IPS specifically predicted response to anti-CTLA4 in ovarian cancer patients in different risk groups, with higher scores resulting in higher response rates. And * represented p < 0.05. **(K)** Violin plots illustrating the responsiveness of different risk groups to six immune checkpoint treatments, where more expression meant better responsiveness to that immune checkpoint treatment. And *** represented p < 0.001. **(L)** The sensitivities of the different risk groups to the six drugs were visualized with box line plots, with a lower IC50 indicating a higher sensitivity to the drug. And *** represented p < 0.001.

The extent of immune infiltration in each group was assessed to comprehensively map immune cells in ovarian cancer. Figure 12A presented a thorough depiction of the distribution of 10 prognostically significant mRNAs and immune infiltration levels assessed by the three methodologies: ESTIMATE, CIBERSORT, and Xcell, illustrating the diverse immune infiltration statuses across different risk groups. The relative abundance of stromal and immune components within the tumor samples, enabling us to assess overall immune infiltration and stromal content differences between risk groups. The allocation of 22 immune cells across different risk categories was subsequently illustrated through a box line plot and heatmap utilizing the CIBERSORT tool (Figures 12B,C). Although M0 macrophages, M2 macrophages, and resting CD4 memory T cells constituted the predominant components of the immunological environment in ovarian cancer, the precise distinctions in distribution between the two groups could not be discerned. Subsequently, we illustrated the correlation between risk scores and 22 immune cell types within the tumor microenvironment of ovarian cancer (Figure 12D). The two primary risk factors were resting dendritic cells and active mast cells, whereas the two most effective protective variables were M1 macrophages and B cell memory. Subsequently, we employed a heatmap (Figure 12E) to analyze the correlations among diverse immune cells, eight risk genes, two protective genes (*HMGN3* and *CXCR4*), risk scores, and prognosis. The risk scores corresponded with the findings of Figure 12D; however, we were focused on the contrasting results about the connection of M1 and M2 macrophages with overall survival (OS). Prognosis exhibited a positive correlation with M1 macrophages, whereas M2 macrophages demonstrated an inverse effect. M2 macrophages were associated with the risk factor *TIMP3*, while M1 macrophages were associated with the protective component *CXCR4*. Previous studies have also used TIMP3 to construct prognostic characteristics and risk stratification, and patients in the high-risk group had a poorer prognosis (Feng et al., 2022). Subsequently, to more effectively analyze the disparities in immune cell expression between the two risk groups, we conducted additional screening for immune cells that were more representative of the ovarian cancer tumor microenvironment (Figures 12F,G). A selection of six representative immune cells was made. It was noteworthy that immune cells positively correlated with risk scores were significantly expressed in the high group, while the low group demonstrated elevated expression of immune cells negatively correlated. Our primary emphasis was macrophages, revealing that M1 macrophages, favorably correlated with prognosis and negatively correlated with risk score, had substantially high expression in the elevated group. This indicated that M1 macrophages served as a protective immune cell type within the ovarian cancer TME. The literature also documented its cancer-inhibiting properties (Gunassekaran et al., 2021).

We calculated the stromal score, immunological score, and ESTIMATE score for both the low and high FTRS groups utilizing ESTIMATE. All values in the low FTRS group were significantly lower than those in the high FTRS group, indicating a greater degree of immune cell infiltration in the high FTRS group (Figure 12H). The group’s response to immunotherapy was then predicted using the Tumor Immune Dysfunction and Exclusion (TIDE), with elevated scores correlating to diminished efficacy of immune checkpoint inhibition medications (Jiang et al., 2018). The TIDE score was significantly higher in the high-risk group compared with the low-risk group (Figure 12I), suggesting that high *FN1* expression significantly impeded the effectiveness of immune checkpoint blockade therapy. Immunophenotypic scores (IPS) of ovarian cancer patients were subsequently used to predict their responsiveness to anti-*CTLA4* therapy (Figure 12J). The data showed that the response rate to anti-*CTLA4* therapy was higher in the high-risk group. Figure 12K depicted six immune checkpoints highly expressed in the high-risk group, including *CD276, CD86, HAVCR2, LAIR1, TNFSF9,* and *TNFRSF14*, which collectively impeded antitumor immune responses. Figure 12L depicted the six drugs with low IC50 values in the high-risk group, suggesting that the high-risk group was more sensitive to these drugs. Overall, the findings suggested that TCs with elevated levels of *FN1* might play a key role in immunotherapy resistance and could serve as a compelling biomarker in predicting survival time in patients with ovarian tumors.

### 3.12 Knockout FN1 *in vitro* studies confirmed its involvement in ovarian cancer

**FIGURE 13:**
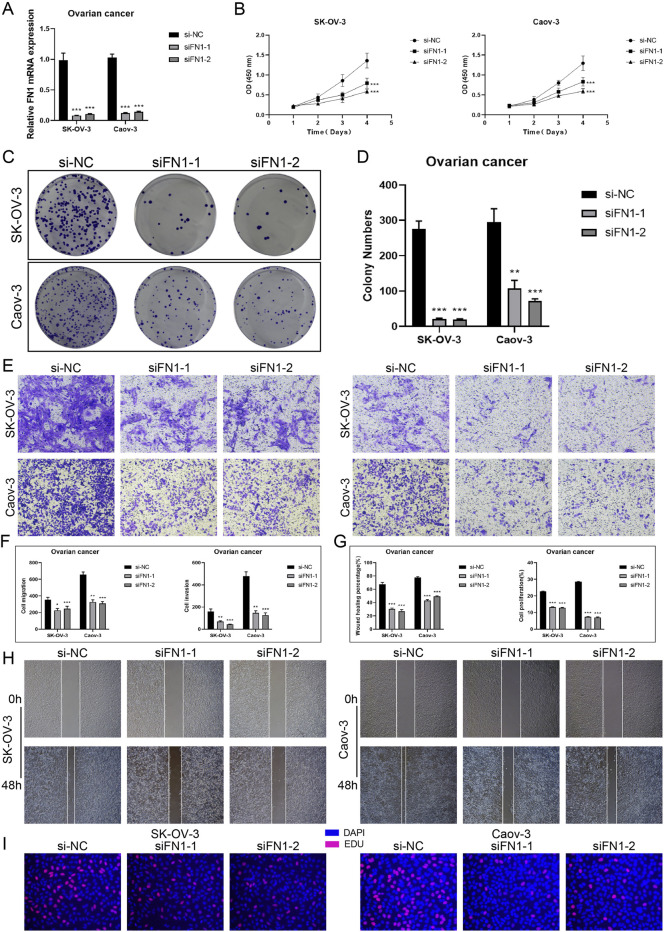
*In vitro* experiments targeting FN1 signaling molecules. **(A)** Knockdown of FN1 significantly reduced mRNA expression levels in both experimental groups (Caov-3 and SK-OV-3). And *** represented p < 0.001. **(B)** CCK-8 testing demonstrated that the viability of TCs was notably reduced following the knockdown of FN1 in comparison to the control group. And *** represented p < 0.001. **(C and D)** Colony formation testing displayed that the knockdown of FN1 significantly reduced the number of colonies in the experimental group. And *** represented p < 0.001. **(E and F)** The migration and invasive abilities of TCs in both experimental groups were inhibited by FN1 knockdown, as demonstrated by the transwell experiments and shown in the quantitative plots. This clarification has been added to all relevant figure notes to enhance reader understanding and interpretability of the results. And * represented p < 0.05, ** represented p < 0.01, *** represented p < 0.001. **(G and H)** The quantitative plots showed a significant reduction in both migration and proliferation abilities of TCs following FN1 knockdown, while the inhibition of TCs migration by FN1 knockdown was indicated by the scratch assays. And *** represented p < 0.001. **(I)** The knockdown of FN1 inhibited the proliferation of TCs was observed by EdU staining test.

The Caov-3 and SK-OV-3 cell lines were utilized in in vitro research to target *FN1* mRNA degradation using siRNA to inhibit *FN1* expression. Initially, mRNA levels were evaluated prior to and after to *FN1* knockdown. Both cell lines exhibited a significant decrease in mRNA expression levels relative to the control group (Figure 13A). Following *FN1* knockdown, the CCK-8 assay demonstrated a significant reduction in TCs viability (Figure 13B). Furthermore, the colony formation assay indicated that *FN1* knockdown inhibited the aggregation of TCs (Figures 13C,D). Furthermore, transwell and scratch assays were employed to evaluate the migratory and invasive capacities of TCs, revealing a significant reduction in both following *FN1* knockdown (Figures 13E–H). This result was also observed by the EdU staining assay (Figure 13I). The aforementioned findings indicated that *FN1* knockdown inhibited ovarian cancer tumor growth by obstructing TCs aggregation, activity, invasion, and migration. This result has provided us with a novel approach for targeted therapy in ovarian cancer inside the clinical setting.

## 4 Discussion

Ovarian cancer exhibited considerable regional and temporal diversity at the molecular, cellular, and anatomical levels. Both innate and learned resistance arose from the varied responses to systemic and surgical interventions due to this complexity. Ovarian cancer is hence highly aggressive and often fatal. Rather than being a singular disease, it has multiple subtypes, each with distinct and evolving molecular profiles that change as the disease advances and is managed. Treatment options were further confounded by the dynamic interactions between cancer cells and stromal components inside the tumor microenvironment, which were essential in facilitating disease progression and modulating the tumor’s response to therapy (Hollis, 2023; Veneziani et al., 2023). The recent therapy strategy for ovarian cancer involves the introduction of immune-related molecularly targeted medicines, which elicit immunostimulatory or immunosuppressive effects alongside their cytostatic and cytotoxic actions against malignant cells (Moniot et al., 2024; Fucikova et al., 2022; Kim et al., 2012; Gritsina et al., 2015). Previous studies have primarily focused on profiling immune cell infiltration and checkpoint expression in bulk RNA-seq data or characterizing tumor heterogeneity through either scRNA-seq or spatial transcriptomics independently. These studies just highlighted immunotherapy potential by profiling immune checkpoint landscapes or T cell functionality, but lacked integrative spatial-functional insight. Our study advances this by combining scRNA-seq, spatial transcriptomics, gene regulatory network analysis (pySCENIC), and intercellular communication analysis (CellChat) to elucidate a comprehensive tumor-immune interaction map, particularly around *FN1*+ tumor cells. This integrative framework surpasses earlier studies by adding spatial resolution and direct experimental validation of therapeutic targets.

We identified six primary cell types, including EPCs, by the analysis of ovarian cancer samples, omental tissue, and normal ovarian tissue. These cell types exhibited an increasing percentage correlated with cancer progression. This illustrated the significance of EPCs in the progression of ovarian cancer. We discovered six kinds of TCs utilizing inferCNV. The C3 *FN1*+ TCs distinguished themselves through their strong association with omental tissue, suggesting their role as metastasis drivers and early facilitators of tumor nesting. This outcome aligned with prior studies indicating that the omentum serves as a pre-metastatic site that facilitates the proliferation of ovarian cancer cells (Li et al., 2023; Lee et al., 2019). Our CytoTRACE research indicated that key stemness markers like as *BMI1* and *CTNNB1* were prevalent in C3 *FN1*+ TCs. These cells were linked to pathways crucial for EMT and tumor proliferation (Banerjee et al., 2017; Wen et al., 2019; Lozneanu et al., 2021). The elevated stemness of this subtype indicates its potential involvement in the first stages of cancer, which may influence the onset of metastasis. The participation of C3 *FN1*+ TCs as an initial subtype was validated by monocle and SlingShot trajectory analyses, which positioned them at the commencement of the developmental pathway. C3 *FN1*+ TCs were posited as essential for understanding carcinogenesis and evolution, derived from developmental trajectories of ovarian to omental cancer tissues. These data suggested that concentrating on the first molecular mechanisms associated with this subtype might be crucial for developing innovative intervention strategies and personalized treatment.

The SCENIC investigation found *ATF3*, *JUND*, and *NR2F1* as significantly active in C3 *FN1*+ TCs. *NR2F1*’s involvement in EMT and chemoresistance, along with *ATF3*’s association with heightened treatment resistance, rendered these factors pivotal in cancer progression (Fu et al., 2021; Li D. et al., 2022; Liu et al., 2023; Rodriguez-Tirado et al., 2022). CellChat research revealed that C3 *FN1*+ TCs were involved in significant intercellular communication, specifically through the *FN1*-*CD44* signaling pathway. Fibroblasts and myeloid cells collaborated to create an environment conducive to cancer proliferation and dissemination (Umansky et al., 2019; Zhang H. et al., 2023; Biffi and Tuveson, 2021). In order to provide additional evidence that there was a connection between tumor subtypes that had a higher FN1 expression and the growth and metastasis of the tumor, we decided to investigate the ST. To translate cell type annotations from scRNA-seq to ST data, tissue sections taken from a patient with ovarian cancer were evaluated using the RCTD approach. According to the findings of the investigation, the C3 *FN1*+ TCs subtype were found to be spatially connected with tumor sites, and their positions were shown to be consistent with the distribution of myeloid cells and circulating *FN1*. Through further analysis utilizing stLearn, it was shown that *FN1*, which was largely expressed by C3 *FN1*+ TCs subtype and fibroblasts, interacted with the receptor *CD44* through paracrine and proximal secretion. This interaction suggested that *FN1* played a crucial role in conveying information between tumors. According to the findings of the study, the C3 *FN1*+ TCs subtype was responsible for the promotion of ovarian cancer through the *FN1*-*CD44* signaling pathway. This route was supported by a paracrine cell-cell communication network that existed inside the microenvironment of the tumor.

The fact that *FN1* had a role in both cell adhesion and migration brought to light the biological potential of this protein as a therapeutic target (Zhang et al., 2017; Zhou et al., 2022). The effective communication between C3 *FN1*+ TCs and immune cells highlighted their ability to employ immune evasion strategies, which might enhance cancer survival and dissemination. Patient stratification could be reliably achieved through the utilization of *FN1*, which might serve as a prognostic biomarker for ovarian cancer. The Caov-3 and SK-OV-3 cell lines utilized in in vitro *FN1* knockdown studies demonstrated dramatic decreases in cell survival, motility, and invasion. These findings indicated the therapeutic potential of targeting *FN1* to disrupt the supportive networks of C3 *FN1*+ TCs, hence reducing tumor development and metastasis while enhancing immuno-precision therapy methods.

The creation of an FTRS model that exhibited robust predictive capability for patient survival, grounded in ten prognostic mRNAs. Upon the creation of an FTRS model, it was observed that patients exhibiting elevated FTRS levels had significantly diminished survival probabilities. This score signified a more aggressive TME that could influence treatment resistance, as it was associated with heightened immune infiltration and immunosuppressive conditions. A higher FTRS score predicted a worse prognosis as well as poorer immunotherapy responsiveness. The immunological landscape of ovarian cancer, wherein specific immune cell types—such as M1 and M2 macrophages—substantially influence patient prognosis and their responsiveness to immunotherapy. Numerous prior papers have demonstrated that M2-type macrophages facilitate immune escape in the tumor microenvironment through diverse mechanisms, resulting in an immunological-hyporesponsive milieu (Ma et al., 2021; Yu et al., 2024; Ning et al., 2023). Immunological checkpoint therapy and personalized treatment strategies might be influenced by immunological escape in high-risk populations. Drug sensitivity testing offered valuable insights into precision medicine for ovarian cancer treatment by identifying potential targeted therapies for high-risk populations. The FTRS model, based on ten prognostic mRNAs, effectively predicts ovarian cancer patient survival by reflecting an immunosuppressive tumor microenvironment linked to poor prognosis, immunotherapy resistance, and potential targets for precision treatment.

Nonetheless, there were some shortcomings in our study. This study utilized only eight tissue samples, potentially failing to represent the diversity of ovarian cancer across different patient populations. Secondly, computational predictions had limitations, and the analysis of immune infiltration patterns, TIDE scores, and immune checkpoint expression were largely based on bioinformatics algorithms and hypothesis generation that had not been experimentally or clinically validated. Although these methods could provide valuable clues, the results needed to be interpreted with caution and future confirmation was still needed in combination with functional experiments and clinical data. Thirdly, although the function of FN1 was validated through *in vitro* experiments utilizing Caov-3 and SK-OV-3 cell lines, these findings may not adequately reflect the *in vivo* tumor microenvironment. Future research should employ *in vivo* models to corroborate these findings.

To sum up, this study concluded that C3 *FN1*+ TCs represented a significant early high-stem subtype that contributed to the progression of ovarian cancer. Their significant role in transcriptional regulation and intercellular communication underscored their importance in influencing the TME. Focusing on *FN1*, a critical target gene, enabled the development of novel immunoprecision therapies aimed at halting tumor growth and enhancing patient prognosis. The distribution of key immune cell types, including M1 macrophages as protective factors and M2 macrophages as risk factors, significantly impacted the immunological landscape of ovarian cancer within the TME. The findings underscored the significance of the TME in shaping disease progression and patient outcomes. The immunologic profile of the high-risk population was correlated with reduced responsiveness to immune checkpoint treatments. This highlighted the necessity for the development of targeted immunotherapies. The creation of novel immunoprecision drugs tailored for ovarian cancer patients might enhance the immune system’s capacity to combat ovarian cancer and increase its efficacy in immunotherapy, resulting in better patient outcomes.

## Data Availability

The original contributions presented in the study are included in the article/Supplementary Material, further inquiries can be directed to the corresponding authors.
