# Supplementary figures and images for: Integrated multi-omics analysis reveals the immunotherapeutic significance of tumor cells with high FN1 expression in ovarian cancer
Source: Front Mol Biosci. 2025 Jun 19;12:1611964. doi: 10.3389/fmolb.2025.1611964 (PMC12221904; doi:10.3389/fmolb.2025.1611964)

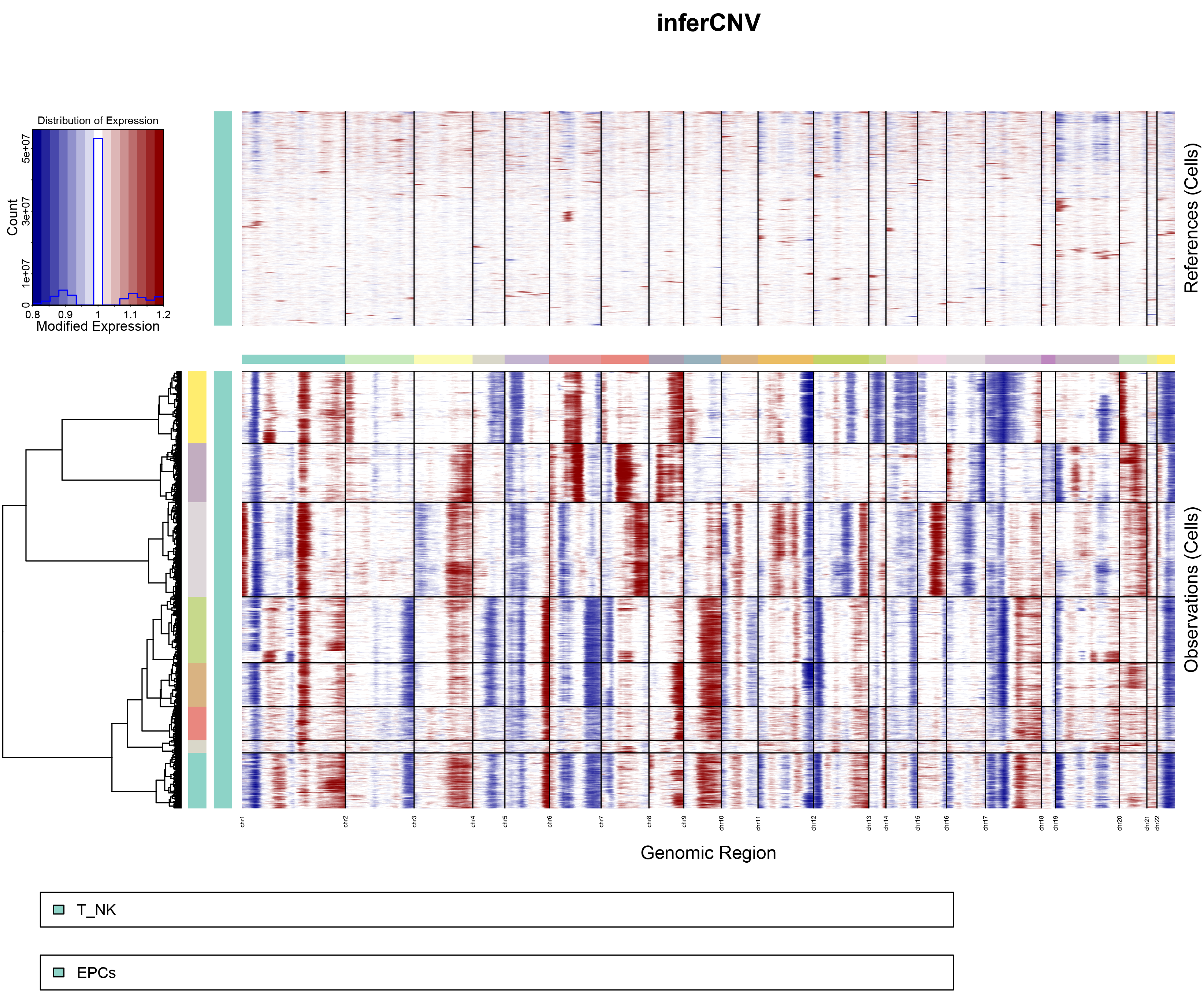

Supplement: Supplementary file 1 [file Image1.TIF]
